# The association between social ties and depression among Asian and Pacific Islander undocumented young adults

**DOI:** 10.1186/s12889-021-11087-y

**Published:** 2021-05-27

**Authors:** Annie Ro, Michelle Kao Nakphong, Hye Young Choi, Alex Nguyen, May Sudhinaraset

**Affiliations:** 1 Department of Health, Society, and Behavior, University of California, Irvine, Irvine, CA USA; 2 Department of Community Health Sciences, University of California, Los Angeles, Los Angeles, CA USA; 3 Department of Social and Behavioral Sciences, Harvard T.H. Chan School of Public Health, Boston, MA USA

**Keywords:** Mental health, Asian Pacific islanders, Social ties, Undocumented immigrants, Young adults

## Abstract

**Background:**

The mental health of Asian and Pacific Islander (API) undocumented young adults has been understudied, despite an increasingly restrictive immigration climate that would ostensibly raise mental health risks. This study examined the role of social ties and depression among API undocumented young adults. We distinguished between two types of social ties, bonding and bridging, and additionally considered the absence of ties (e.g. isolation).

**Methods:**

We used primary data collected among 143 API undocumented young adults. We first identified correlates for each type of social tie and then examined the association for each measure with depression.

**Results:**

Higher levels of bonding and bridging ties were associated with lower odds of a positive depression screen. In contrast, isolation was associated with higher odds of a positive depression screen. There were no significant associations between total social ties and depression.

**Conclusions:**

Our findings suggest that both bonding and bridging ties are important factors in the mental health of API undocumented young adults. Factors that facilitate these types of ties, such as DACA, can be effective interventions for improving mental health among this population.

**Supplementary Information:**

The online version contains supplementary material available at 10.1186/s12889-021-11087-y.

## Background

Approximately 15% of the 11 million undocumented immigrants in the United States are young adults between 16 and 24 years of age [1]. Within the population of undocumented young adults, Asians and Pacific Islanders (APIs) have been understudied. Despite making up 14% of the undocumented population [2] and 25% of the undocumented college student population [3], we know little about how API undocumented immigrants are faring in their mental health. This knowledge gap is especially concerning in light of the increasingly restrictive immigration climate. Federal policy threats to Deferred Action for Childhood Arrivals (DACA), the public charge rule, bans on refugees/asylees from specific countries, family separation at the border, and increased federal immigration enforcement activities have pushed undocumented immigrants further into the shadows, potentially leading to more social isolation and mental health problems [4].

### “Illegality” and mental health among undocumented young adults

“Illegality”, that is, the legal and social processes that make immigration status consequential in everyday life, target the quotidian activities of undocumented immigrants, restrain their economic and social mobility, and produce strain and stress [5]. A review of mental health status among Latino undocumented immigrants found widespread isolation, stress, depression, and vulnerability [6]. The experience of “illegality” is unique for young adults, as they have spent critical developmental periods in the US. Undocumented young adults are often not aware of their legal status until late adolescence, are educated in the US, and have certain legal protections not available to older immigrants, such as DACA [7]. DACA was an Obama-era Executive Order that deferred deportations and provided work permits to undocumented young adults who met certain provisions.

There are several sources of mental health strain owing to the unique illegality experience of undocumented young adults [8]. Those who go on to higher education receive little family financial support and may even be expected to contribute financially to the household [9]. They also face barriers to milestones that are taken for granted among their peers, such as receiving a driver’s license, applying to college, or obtaining employment. Their status also brings stigma and shame, which many internalize, resulting in social isolation and trauma [10]. The current immigration climate serves as a context in which undocumented young adults have experienced a higher sense of fear of deportation, not only just for themselves, but particularly for their parents and other family members [11].

For API undocumented young adults, experiences of illegality may be more singular still. On the one hand, API undocumented immigrants in general have higher socioeconomic status than Latino undocumented immigrants [12], which can alleviate some of the financial strain and responsibility that young adults feel. APIs may experience less of the racialized stigma of undocumented status, which is primarily applied to Latino populations [13].

On the other hand, the smaller size of the undocumented API population may intensify stigma within their own co-ethnic communities, restricting undocumented young adults’ social networks and resulting in social isolation [14]. Additionally, there may be fewer resources for the API undocumented because of their small population size, leaving API undocumented young adults less aware about how to access services, such as in higher education settings. For example, API application rates for DACA are significantly lower compared to Latino groups (21% vs. 77%, respectively) potentially due to fewer community resources for applying [15]. API undocumented young adults may not associate with the larger Latino undocumented population, isolating them further from potential resources  [16].

### Social ties and mental health

Collectively, these experiences further push undocumented young adults into the shadows as they keep their status a secret from their social networks and disengage from potential resources and opportunities [17]. This isolation is especially concerning in light of research that highlights the importance of social ties in mental health [18, 19]. Past studies find that social isolation, smaller social networks, and lower perceived social support are associated with increased depressive symptoms across the life course from adolescence [20] to the elderly [21]. Researchers have suggested that social relationships may directly affect mental health through promoting positive health behaviors, such as increased exercise, or indirectly affect mental health through a stress-buffering model in which perceived social support is thought to buffer the effects of stress by enhancing coping strategies [18, 22]. These two mechanisms are not mutually-exclusive.

While the link between social ties, social relationships, and mental health is well-established, less is known about the social ties of immigrants, particularly undocumented young adults, and the extent of their social networks. Previous work has found high mental health needs among API undocumented young adults and has underscored the importance of social ties in shaping their well-being [10]. In focus group discussions and in-depth interviews, API undocumented young adults expressed extreme inter-ethnic distrust among Asian immigrant communities and even extended family members after being exploited or misled on account of their own or their parents’ documentation status. Compounding issues of mental health issues and trauma, many were deterred from seeking services by their family members due to concerns with their legal status. Their precarious legal status and associated stigma prevented them from leveraging social relationships to seek out important resources for their health.

Two particularly important types of social ties are bonding and bridging social capital [23, 24]. First, “bonding” social capital refers to the social resources derived from members of a network with similar characteristics such as class, race/ethnicity. While this may provide a sense of solidarity and support, it may also reproduce social disadvantage depending on the resources and norms in the community. On the other hand, “bridging” social capital refers to the resources accessed across networks that cross class, race/ethnicity, or other social characteristics. Bridging social capital is generally associated with better health outcomes, including increased access to health information [25]. Bridging social capital is linked to the exposure to and development of new ideas, values, and perspectives [24]. While bonding social ties are typically centered at the micro-level including close individuals, families, and households, bridging social ties occur at the macro-level, such as across organizations. Bridging social capital is typically associated with better employment and income [26] and self-rated health [27] compared to bonding social capital, while other studies find a protective effect for both types of social ties [28]. To our knowledge, no studies have quantitatively examined the social ties for undocumented young adult Asian and Pacific Islanders, despite qualitative studies suggesting high levels of mental health problems and social isolation.

### Study overview

In this study, we utilize primary data to examine the association between social ties and mental health among API undocumented young adults. Given the difficulty in identifying a sizeable sample of Asian undocumented young adults in secondary data sources, primary data collection is one of the most efficient ways to study this hard-to-reach population. We first identify correlates of having social ties within API undocumented young adults in order to identify who is likely to have which type of social tie (bonding, bridging, or no ties). We then examine the relationship between different types of social ties and mental health status. We specifically consider whether having bonding ties, bridging ties, and the absence of ties (i.e., isolation) in different help-seeking scenarios lower or raise the likelihood of having depression. We expect a divergence between bonding and bridging ties. We hypothesize that reporting more bridging ties will be associated with lower depression, as these ties produce support across different networks and institutions, whereas bonding social ties and depression will have a null or minimally negative association given the existing empirical literature. Finally, we expect that the absence of social ties, which we conceptualize as isolation, to be associated with higher depression.

## Methods

### Sample

This paper uses data from the 2019 BRAVE (Building community Raising API Voices for health Equity) California Asian Pacific Islander Immigrant Social Networks and Health Study. This survey data was collected online between June and August 2019 using Qualtrics. We employed a community-engaged approach by recruiting a Community Advisory Board (CAB) and community interns to co-develop study materials and facilitate participant recruitment [29]. As with previous iterations of the BRAVE Study and other studies involving undocumented young adults, this approach allowed us to more effectively engage the community in determining appropriate domains of interest, co-creating and pilot-testing new items for face validity, and exchanging knowledge about public health research methods and network-based assets for recruitment efforts [10].

We used convenience sampling to recruit participants through school resource centers, including undocumented student services centers; community-based organizations; and social media campaigns, with the help of our CAB and student interns. Eligible individuals were: 1) Asian or Pacific Islander; 2) undocumented with or without DACA; 3) between the ages of 18–31; 4) enrolled in a private college or university, California Community College, California State University, and/or University of California campus after June 15th, 2012, when DACA was first enacted; and 5) able to take a 30-min online survey in English. Upon completion of the survey, all survey participants who submitted valid email addresses entered a raffle for a $100 electronic gift card and the first 180 participants also received a $5 electronic gift card. Our project was approved by the [withheld].

There were 209 participants who completed the survey. We used list-wise deletion on our full set of analytic variables in our regression models to get an analytic sample of 143. We conducted multiple missing checks to determine if our results were biased by the dropped observations. There were no statistically significant differences between the 66 dropped respondents from the analytic sample in regards to age, gender, employment status, mother’s education, or student status, leading us to conclude our data was not missing at random (MAR) and was not appropriate for multiple imputation.

### Study measures

#### Depression

The DSM-V characterizes depression as a multidimensional mood disorder that includes negative emotion, an absence of positive emotions (i.e., anhedonia), physical symptoms (i.e., weight loss, fatigue), inability to concentrate, or recurrent thoughts of death or suicide [30]. These symptoms have to present for at least two weeks and cannot be due to another medical condition. We use the Center for Epidemiological Studies-Depression 10 (CES-D 10) scale to measure depression, which has been shown to be a valid screening tool for major depressive order. This is a shortened scaled from the Center for Epidemiological Studies- Depression. This scale has been previously used among API young adults [31–33]. Composite scores were calculated from the 10-item scale by scoring the Likert-scale response options “Rarely,” “Sometimes,” “Occasionally,” and “All of the time” from 0 to 3 respectively. We dichotomized the variable to indicate those above and under the clinical depression cut-off score of 10 or higher [34].

#### Social ties

Survey respondents answered questions regarding experiences applying to and attending college. These responses were used to generate four social ties scores: total social ties, bonding ties, bridging ties, and absence of social ties. Respondents identified one person they received support from in 14 different help-seeking scenarios such as accessing campus services, seeking health services, and navigating the immigration system and DACA applications, etc. The mutually exclusive response options were parents, other family members (i.e. siblings), friends, school counselors, school teachers, health providers or professionals, lawyers, the Internet, other, or no one. For the total social network score, we summed the number of items across the 14 scenarios in which respondents identified someone they could rely on for support (i.e. any response that was not “the Internet” or “no one”). The total social ties score ranged from 0 to 14; higher scores indicate a greater number of scenarios in which respondents report utilizing social ties for support.

We conceptualized bonding ties as those from family, friends, and social contacts. We summed the number of items across the 14 scenarios in which respondents identified their parents, other family members, or friends as someone they could rely on for support in these help-seeking scenarios. We conceptualized bridging ties as those from formal contacts. To calculate a bridging ties score, we summed the number of items across the 14 scenarios which respondents identified support from school counselors, school teachers, health providers or professionals, lawyers, or others. To calculate an “absence of social ties” score, we took the sum of items in which respondents indicated they either had no source of support or they turned to the Internet for information in help-seeking scenarios (Cronbach’s alpha for total social ties score = 0.89; bonding = 0.79; bridging = 0.82, no social ties = 0.89).

The social ties measure was developed for this study but mirrors others that have asked about the presence of ties in different scenarios [35]. We created dichotomous variables for each type of social ties score (i.e. total, bonding, bridging, absence of social ties), coding scores at or above the median as ‘high’ and scores below the median as ‘low.’ The median number of total social ties was 10, median number of bonding ties was 3, median bridging ties was 3, and the median number of no reported social ties was 4. For example, a high bonding score was indicated by reporting reliance on family or friends for 3 or more help-seeking scenarios and a low bonding score was indicated by reporting reliance on family or friends for less than three help-seeking scenarios.

#### Covariates

Covariates included age, gender, highest level of education, type of higher education institution, current employment status, and DACA status. Age was stratified into 2 categories: 18–25 and 26 and older to roughly distinguish between college-aged respondents and older. Gender was separated between males and females. Highest level of education categories were High School or Less (including those who responded they had no school, less than high school, or high school or equivalent); Some College (including those who responded they had attended some college or a 2-year community college institution); and College/University or Higher, (including those who responded they had attended a 4-year college, graduate, and/or professional school). Type of higher education institution included the categories not a student, those who attended a Community College, a California State University (CSU), a University of California institution (UC), or a private institution. While Community Colleges, CSUs, and UCs are all public college systems within California, all operate independently with varying amounts of resources for each system. Employment status was separated into those who were currently employed, including part- or full-time, versus those not currently employed. DACA status was a dichotomous variable, categorizing respondents who were current DACA recipients compared to those who either were not recipients, pending renewal, had their application denied, or who never applied for the program.

### Analyses

We examined sociodemographic characteristics, a positive depression screen (as indicated by the CES-D 10), and reported social ties of the sample. We explored distributions of categorical variables as well as means and standard deviations for continuous variables. We examined bivariate associations between 1) demographic factors and social ties scores and 2) social ties and depressive symptoms using chi-square tests and t-tests. Multivariate logistic regression analyses were then performed using the dichotomous social ties variables to further examine the relationship between social ties and depression, adjusting for the covariates described above.

We plotted Pearson’s standardized residuals against predicted probabilities and found that there were no extreme or influential observations. We used link tests for model specification and tests indicated that factors were meaningful and there was no evidence of specification error. Hosmer-Lemeshow goodness-of-fit tests indicated that models were well calibrated. For statistical significance, alpha level was set at 0.05. All analyses were performed using Stata/SE 15.1.

## Results

### Descriptives

Table 1 provides the descriptive characteristics of the sample. The majority of participants were between ages 18–25 (80.4%). Male participants comprised roughly over half of our sample (54.5%). We observed a relatively even distribution of socioeconomic status among our participants, in which 27.3% attained a high school level of education of less, 35.7% attended some college, and 37.1% graduated with a college/university degree or higher. Among those enrolled in an undergraduate school at the time of the study, the largest proportion attended a school in the California State University (CSU) system (37.1%), followed by a school in the University of California system (33.7%). Over two-thirds (67.1%) of participants stated they were working full-time or part-time during the study. A similar percentage of the participants were DACA recipients. Chinese (30.1%) and Korean (21.7%) respondents made up more than half of the sample.

**Table 1:** Descriptive Characteristics of Sample (*n* = 143)

Variable	N or mean	% or (SD)
Age	23.6	(3.7)
Age categories		
18–25 years	115	80.4%
26 years or older	28	19.6%
Gender		
Female	65	45.5%
Male	78	54.5%
Highest level of education		
High school or less	39	27.3%
Some college	51	35.7%
College/University or higher	53	37.1%
Currently enrolled as an undergraduate and institution type		
Not enrolled as an undergraduate	16	11.2%
Community College	30	16.9%
California State University	66	37.1%
University of California	60	33.7%
Private	22	12.4%
Currently employed (FT or PT)		
No	47	32.9%
Yes	96	67.1%
Ethnicity		
Chinese	39	30.1%
Korean	21	21.7%
Filipino	12	8.4%
Vietnamese	9	6.3%
Other	52	36%
DACA recipient		
No	46	32.2%
Yes	97	67.8%
Social Ties Total Score (range 0–14)	8.77	4.29
Bonding Ties Score (range 0–14)	3.8	2.93
Bridging Ties Score (range 0–14)	4.08	3.29
No Social Ties Score (range 0–14)	5.1	4.2

Overall, the mean social ties total score was 8.77 (SD 4.29). The mean bonding ties score was 3.8 (SD 2.93), while the mean bridging ties score was slightly higher, at 4.08 (SD 3.29). The mean score for the absence of social ties was 5.1 (SD 4.2), meaning that, on average, respondents could not identify someone they could rely on for support in 5 out of 14 scenarios.

### Bivariates analyses

#### Correlates of social ties

We compared those with high total social ties scores (reporting reliance on social ties for 10 or more questions) with those with low scores across sociodemographic characteristics (Table 2). On average, those with a high number social ties scores tended be younger (mean age = 22.74, SD 1.96) than those with lower scores (mean age = 24.38, SD 4.72) (*p* = 0.007). Relatedly, those with a low number of social ties scores had a larger proportion of participants 26 years and older compared to high scores (30.4% vs. 9.5%, *p* = 0.002). Those with lower scores also tended to have higher educational attainment than those with higher scores (*p* = 0.006). More participants with high social ties scores were also current students (95.9% vs. 81.2%) and had a greater proportion in California State Universities (43.2% vs. 26.1%) but lower proportion in private institutions (8.1% vs. 13.0%) than those with low scores (*p* = 0.028). Notably, a majority of those with high total social ties scores were DACA recipients (90.5%), whereas less than half (43.5%) of those with low total social ties scores reported receiving DACA (*p* < 0.001). These two groups did not significantly differ with respect to gender, or employment status.

**Table 2:** Demographic characteristics by social ties scores

	Total Social Ties				
	Low (below median)	High (at or above median)			
Variable	N or Mean	% or (SD)	N or Mean	% or (SD)	***p***-value
**Total**	69		74		
Age (continuous)	24.38	(4.72)	22.74	(1.96)	0.007
Age categories					
18–25 years	48	69.6%	67	90.5%	0.002
26 years or older	21	30.4%	7	9.5%	
Gender					
Female	36	52.2%	29	39.2%	0.119
Male	33	47.8%	45	60.8%	
Highest level of education					
High school or less	12	17.4%	27	36.5%	0.006
Some college	23	33.3%	28	37.8%	
College/University or higher	34	49.3%	19	25.7%	
Currently enrolled as an undergraduate in a Public or Private California college					
Not student	13	18.8%	3	4.1%	0.028
Community College	12	17.4%	14	18.9%	
California State University	18	26.1%	32	43.2%	
UCs	17	24.6%	19	25.7%	
Private	9	13.0%	6	8.1%	
Currently employed (FT or PT)					
No	21	30.4%	26	35.1%	0.550
Yes	48	69.6%	48	64.9%	
DACA recipient					
No	39	56.5%	7	9.5%	< 0.001
Yes	30	43.5%	67	90.5%	

#### Social ties and depression

Of the sample, 62.2% of participants screened positive for depression (Table 3). Bivariate analyses of social ties scores and depression screen indicated that there were statistically significant bivariate associations between all types of social ties and positive depression screens. For example, 39.3% of those with high total social ties screened positive for depression compared to 72.2% of those with low total social ties scores (p < 0.001). Similar bivariate associations were found for bonding and bridging ties. For bonding ties, 52.8% of those who reported bonding ties at or above the median screened positive for depression, while 81.5% of those reporting less than the median number of bonding ties screened positive (*p* = 0.001). For bridging ties, 53.9% of high scoring participants screened positive for depression, compared to 79.6% of low scoring participants (p = 0.002). Finally, an inverse relationship was observed for no social ties scores and positive depression screens: 74.2% of those who reported a high number of no social ties screened positive for depression, compared to 40.7% of those who reported a low number (p < 0.001).

**Table 3:** Depression Screen Results by Total, Bonding, Bridging, and “No One” Social Ties Scores

	Screened positive for depression				
	No (CESD < 10)		Yes (CESD ≥ 10)		***p-***value
Indicator	N	%	N	%	
Total number in group	54	37.8%	89	62.2%	
Social network score					
Low (below median)	15	27.8%	54	60.7%	< 0.001
High (at or above median)	39	72.2%	35	39.3%	
Bonding score					
Low (below median)	10	18.5%	42	47.2%	0.001
High (at or above median)	44	81.5%	47	52.8%	
Bridging score					
Low (below median)	11	20.4%	41	46.1%	0.002
High (at or above median)	43	79.6%	48	53.9%	
No Social Ties score					
Low (below median)	32	59.3%	23	25.8%	< 0.001
High (at or above median)	22	40.7%	66	74.2%	

### Multivariate analyses

Results from multivariate logistic regression analyses indicated that higher scores for bonding and bridging ties were protective at a statistically significant level against screening positive for depression after adjusting for covariates (Table 4). Higher bonding ties scores were associated with 66% decreased odds of screening for depression compared to lower scores (aOR = 0.34, 95%CI: 0.14, 0.85). Bridging ties scores at or above the median were associated with 65% decreased odds of screening positive for depression (aOR = 0.35, 95%CI: 0.15, 0.81), compared to those reporting bridging ties below the median. In addition, those who scored high on the absence of social ties had higher odds of a positive depression screen compared to those scoring below the median (aOR = 2.92, 95%CI: 1.25, 6.82). Total social ties scores at or above the median was associated with a decrease in odds of screening positive for depression (aOR = 0.45, 95%CI: 0.18, 1.11), compared to scores below the median, however, this association with not statistically significant.

**Table 4:** Logistic Regression Results for Odds of Positive Depression Screen by Total, Bonding, Bridging, and “No One” Social Ties Scores

	Screened positive for depression (CESD-10 Score 10+)	
Indicators	aOR	95%CI
Social Capital Total Score (ref. Low)		
High	0.45	(0.18, 1.11)
Bonding Capital Score (ref. Low)		
High	0.34*	(0.14, 0.85)
Bridging Capital Score (ref. Low)		
High	0.35*	(0.15, 0.81)
No Social Ties Score (ref. Low)		
High	2.92*	(1.25, 6.82)

*** *p* < 0.001, ** *p* < 0.01, * *p* < 0.5

Models adjusted for age group (ref: < 25 years), gender (ref: female), highest level of education (ref: high school), currently in school (ref: no), employment status (ref: no), and DACA status (ref: no)

### Sensitivity analyses

We conducted the analyses described with alternative coding schemes: dichotomizing the social ties scores at the mean and continuous measures for social ties (Supplemental Tables 1–2). These results produced qualitatively similar results: having more social ties and having more bonding and bridging ties across various help-seeking scenarios was associated with lower likelihood of a positive depression screen (or higher number of depressive symptoms), while more scenarios without social ties was associated with a higher odds of positive depression screen. While each iteration did not produce the same statistically significant results, the overall patterns were the same and we suspect the small sample contributed to the lack of statistical significance for some of the associations.

## Discussion

In this paper, we examined the role of social ties in depression among a sample of API undocumented young adults. To our knowledge, this is one of the only data sources of API undocumented young adults that contains this information. First, a high proportion of the sample screened positive for depression, with over 62% of the sample. This is substantially higher than the estimated 8.8% prevalence of major depression among the general population using the longer version of our scale [36]. We find that those younger than 25 years of age and DACA recipients had more social ties, an important finding when considering who is at risk for social isolation and low social support. We acknowledge that the proportion of DACA recipients was higher than expected for API population generally [37], and would therefore expect these results to be conservative. Yet other studies have also found that DACA promotes social belonging through increased access to opportunities, including employment and education networks [9]. Moreover, the legal protections conferred by DACA empower young undocumented API to engage in community activism, which may contribute to increased social ties [7]. The Biden Administration’s Executive Order to preserve and fortify DACA will likely bolster the mental health of DACA-eligible immigrants and their families. Past studies have found that during periods of DACA uncertainty, young undocumented immigrants report worse self-rated health [38]. These findings highlight the important role that DACA may potentially play in integrating young adult immigrants and improving broader mental health outcomes.

When we considered the link between specific types social ties and mental health, we found that respondents who reported having a high number bridging social ties in different help-seeking scenarios had lower odds of a positive depression screen. Having more bridging ties may have fostered access to resources on-campus and in responding immigration concerns, alleviating mental strain. Contrary to out hypothesis we found having a high number of bonding ties was also associated with lower odds of a positive depression screen. This goes against other research that has found that bonding social ties was not associated with increased employment [26] and self-rated health [27], whereas bridging social capital facilitated both increased employment opportunities and health. While research has found that bonding ties also produce emotional burden while simultaneously offering important social support [39], in our sample, bonding ties could symbolize the available emotional support for our respondents, which can serve as a protective factor in their mental health.

The absence of social ties across the various help-seeking scenarios, which we characterize as being isolated, was also significantly associated with higher odds of a positive depression screen. Other studies among Latino adolescents have found that social isolation was associated with suicidal ideation [40]. The context of social isolation for Asian undocumented young adults is complex, intersecting with issues related to past experiences of inter-ethnic conflict, workplace exploitation, parental documentation status, discrimination, and community stigma associated with being undocumented [10].

One unexpected finding was that the total number social ties was not associated with a positive depression screen. While the odds ratio was in the expected direction, it did not reach statistical significance. There may be several reasons for this finding. First some social ties for API undocumented young adults are stressful and can produce mental strain [10]. For most respondents, social ties, whether from bonding or bridging sources, seem to bolster their mental health and reduce their risk for a positive depression screen. Yet for some respondents, having more ties may create more burden and strain on their mental health. Alternatively, these respondents may rely heavily on their social ties to navigate through different scenarios because of their high mental health needs.

Our data contains some limitations. Because our data is cross-sectional, we cannot ascertain whether a higher number of social ties facilitated better mental health or whether poor mental health reduced the likelihood that one was able to make and utilize social ties meaningfully. We do not know whether our sample is representative of all API undocumented young adults. For instance, well over 50% of our sample had DACA, while other estimates put the actual percentage of DACA recipients among eligible APIs at a much lower rate [37]. This may potentially be due to the study sample, which focused on young adults who are currently or recently graduated from school and therefore more likely to qualify for DACA. Additionally, our sample comes from an internet-based survey, and therefore, those without access to internet may be under-represented in the study. All of these limitations would likely bias our results to be more conservative. The survey was also offered in English; while this study focused on young adult undocumented, there is a possibility that those with limited English proficiency may be under-represented in the survey. We acknowledge that the API population is heterogeneous but we could not control for country of birth, nor could we examine specific ethnic subgroups because of the small sample sizes. Also, while we use the terminology “Asian and Pacific Islander”, the vast majority of our respondents were Asian American and we may not capture the unique needs of Pacific Islanders. Moreover, this study includes a crude measure of bonding and bridging social ties. While there is no uniform measure of bonding and bridging social capital, others have operationalized measures that include measures related to interpersonal trust, feelings of closeness, and reciprocity [28, 41]. Future studies should assess not only who undocumented young adult immigrants are tied to, but also the nature and quality of social ties.

## Conclusions

The immigrant policy climate has been increasingly hostile to immigrants in recent years, including the tenuous nature of DACA. Consequently, young adult undocumented immigrants may be further pushed into the shadows and face mental health problems. This study has a number of policy and programmatic recommendations. First, schools should provide on-site mental health counselors trained to work with young undocumented immigrants. Schools also have a role to play in providing an environment that promotes social integration among undocumented students, including through undocumented student programs. Because undocumented API students may be in the minority in many schools, targeted outreach may be conducted linking alumni and specific resources. This study highlights the importance of bridging social ties, or ties with formal networks in improving mental health. This suggests that teachers, counselors, healthcare providers, and other institutions have a role to play in providing information and support to young adult APIs. Additionally, this study highlights the importance of DACA in improving social ties and potentially mental health. Policy-makers should expand inclusive policies that protect the rights of young adult undocumented immigrants.

## Supplementary Information

**Additional file 1 Supplemental Table 1**. Logistic Regression Results for Odds of Positive Depression Screen by Total, Bonding, Bridging, and “No One” Social Ties Scores (dichotomized at the mean). **Supplemental Table 2**. Logistic Regression Results for Odds of Positive Depression Screen by Total, Bonding, Bridging, and “No One” Social Ties Scores (continuous scores).

## Data Availability

The datasets used and/or analyzed during the current study are available from the corresponding authors on reasonable request.
