# Supplementary material for: The association between social ties and depression among Asian and Pacific Islander undocumented young adults
Source: BMC Public Health. 2021 May 27;21:994. doi: 10.1186/s12889-021-11087-y (PMC8157637; doi:10.1186/s12889-021-11087-y)
Supplement: Supplementary file 1 — Additional file 1 Supplemental Table 1. Logistic Regression Results for Odds of Positive Depression Screen by Total, Bonding, Bridging, and “No One” Social Ties Scores (dichotomized at the mean). Supplemental Table 2. Logistic Regression Results for Odds of Positive Depression Screen by Total, Bonding, Bridging, and “No One” Social Ties Scores (continuous scores). [file 12889_2021_11087_MOESM1_ESM.docx]

**Supplemental Table 1. Logistic Regression Results for Odds of Positive Depression Screen by Total, Bonding, Bridging, and “No One” Social Ties Scores (dichotomized at the mean)**

|  | **Screened positive for depression (CESD-10 Score 10+)** | |
| --- | --- | --- |
| **Indicators (above vs. below mean)** | **aOR** | **95%CI** |
| Social Capital Total Score (ref. Low) |  |  |
| High | 0.45 | (0.18, 1.11) |
| Bonding Capital Score (ref. Low) |  |  |
| High | 0.35* | (0.15, 0.81) |
| Bridging Capital Score (ref. Low) |  |  |
| High | 0.84 | (0.39, 1.81) |
| No social ties Score (ref. Low) |  |  |
| High | 2.12 | (0.86, 5.21) |

**Supplemental Table 2. Logistic Regression Results for Odds of Positive Depression Screen by Total, Bonding, Bridging, and “No One” Social Ties Scores (continuous scores)**

|  | **Screened positive for depression (CESD-10 Score 10+)** | |
| --- | --- | --- |
| **Indicators** | **aOR** | **95%CI** |
| Social Capital Total Score | 0.87* | (0.78, 0.97) |
| Bonding Capital Score | 0.96 | (0.84, 1.10) |
| Bridging Capital Score | 0.91 | (0.81, 1.03) |
| No one Score | 1.17** | (1.04, 1.30) |

*** p<0.001, ** p<0.01, * p<0.5

Models adjusted for age group (ref: <25 years), gender (ref: female), highest level of education (ref: high school), currently in school (ref: no), employment status (ref: no), and DACA status (ref: no)
